# The program of complex differentiated medical and psychological rehabilitation of suicidal behavior in dementia

**DOI:** 10.1192/j.eurpsy.2021.2190

**Published:** 2021-08-13

**Authors:** I. Mudrenko, O. Potapov, E. Alswaeer

**Affiliations:** Department Of Neurosurgery And Neurology, Sumy State University, Sumy, Ukraine

**Keywords:** dementia, suicidal behavior, medical and psychological rehabilitation

## Abstract

**Introduction:**

The course of dementia is accompanied by aggression, wandering, agitation, sexual and eating disorders, suicidal behavior (SB).

**Objectives:**

Develop and approbate a program of medical and psychological rehabilitation (MPRP) SB in patients with dementia.

**Methods:**

It were treated 199 patients with SB in dementia of which 107 get cured according to the developed programs and 92 people received traditional treatment.

**Results:**

The program of MPRP combines pharmacotherapy, psychotherapy psychoeducation, psychological training and developed taking into account the mechanisms and predictors of SB. The program included phases: diagnostic phase, phase of active intervention, psychoprophylactic phase. Pathogenetic treatment of dementia was performed with acetylcholinesterase inhibitors and / or NMDA-receptor blockers for 4-6 months. Patients with dementia with a depressive mechanism of SB were additionally prescribed antidepressants from the class of SSRIS for 3-4 months; with the psychotic mechanism of SB – atypical neuroleptics (risperidone, quetiapine) for 2-3 months. The system of psychotherapeutic and psychosocial intervention included rational and family psychotherapy, cognitive training, self-care training and psychoeducation for patients with the cognitive mechanism of SB – art therapy and family psychotherapy, communicative trainings and psychoeducation for patients with the depressive mechanism of SB; crisis psychotherapy and art therapy, social skills training and psychoeducational classes for patients with a psychotic mechanism. Improvement of mental state and reduction of symptoms of SP were diagnosed in 72.9% of patients, and after the use of traditional forms of prevention – only in 40.2% (DC=2.58; MI=0.43, p>0.001).

**Conclusions:**

The results of approbation MPRP program in SB testify to its effectiveness.

**Disclosure:**

No significant relationships.

